# Counteracting Immunosenescence—Which Therapeutic Strategies Are Promising?

**DOI:** 10.3390/biom13071085

**Published:** 2023-07-06

**Authors:** Christoph Hieber, Stephan Grabbe, Matthias Bros

**Affiliations:** 1 Department of Dermatology, University Medical Center of the Johannes Gutenberg-University Mainz, Langenbeckstraße 1, 55131 Mainz, Germany; chieber@uni-mainz.de (C.H.); stephan.grabbe@unimedizin-mainz.de (S.G.); 2 Institute of Molecular Biology (IMB), Ackermannweg 4, 55128 Mainz, Germany

**Keywords:** immunosenescence, inflammaging, senescence-associated secretory phenotype, nanodrug, nanovaccine

## Abstract

Aging attenuates the overall responsiveness of the immune system to eradicate pathogens. The increased production of pro-inflammatory cytokines by innate immune cells under basal conditions, termed inflammaging, contributes to impaired innate immune responsiveness towards pathogen-mediated stimulation and limits antigen-presenting activity. Adaptive immune responses are attenuated as well due to lowered numbers of naïve lymphocytes and their impaired responsiveness towards antigen-specific stimulation. Additionally, the numbers of immunoregulatory cell types, comprising regulatory T cells and myeloid-derived suppressor cells, that inhibit the activity of innate and adaptive immune cells are elevated. This review aims to summarize our knowledge on the cellular and molecular causes of immunosenescence while also taking into account senescence effects that constitute immune evasion mechanisms in the case of chronic viral infections and cancer. For tumor therapy numerous nanoformulated drugs have been developed to overcome poor solubility of compounds and to enable cell-directed delivery in order to restore immune functions, e.g., by addressing dysregulated signaling pathways. Further, nanovaccines which efficiently address antigen-presenting cells to mount sustained anti-tumor immune responses have been clinically evaluated. Further, senolytics that selectively deplete senescent cells are being tested in a number of clinical trials. Here we discuss the potential use of such drugs to improve anti-aging therapy.

## 1. Introduction

Physiological aging is accompanied by impaired functional activity of the immune system, termed immunosenescence [1]. Immunosenscence is characterized by alterations in the frequencies of immune cell subpopulations [2], an attenuated responsiveness of innate immune cells towards danger signals [3], impaired functional activities of antigen-presenting cells (APCs) [4], and adaptive immune cells comprising B cells [5] and T cells [6]. The extent of immunosenescence is mimicked and strongly influenced by chronic (viral) inflammations [7] as exemplified especially by cytomegalovirus (CMV) [8]. One hallmark of immunosenescence is persistent low-level inflammation, termed inflammaging [9], which is associated with a senescence-associated secretory phenotype (SASP), especially of innate immune cells like macrophages [10], but also by p^16Ink4a^+ senescent cells that expand age-dependently due to an attenuated turnover [11] and also arise in response to various stress factors [12]. The adaptive immune system is characterized by lower levels of naïve T cells and B cells but elevated numbers of memory T cells [13] and B cells [14]. Whereas inflammaging is considered to contribute to the induction of (auto)inflammatory diseases [15], the functional impairment of antigen-presenting cells (APCs) and of adaptive immune cells has been acknowledged as the main cause of the limited success of conventional vaccinations in elderly [16].

Improvements in medical care have enabled a strong increase in life expectancy [17]. In order to promote healthy aging, it will be necessary to develop anti-aging strategies that act on the level of the immune system. To date, caloric restriction [18] and dietary agents that elevate autophagy [19], e.g., by enhancing sirtuin (SIRT)1 activity [20] and inhibiting phosphoinositide 3-kinase (PI3K)/mammalian target of rapamycin (mTOR) signaling [21], respectively, have been reported to revert aspects of immunosenescence [22]. Hence, the delivery of such drugs in a cell type-directed manner may be decisive for healthy aging. For this, cell type targeting nanocarriers (NCs), which so far have been developed mainly for anti-tumor (immuno)therapy [23], might be suitable. Likewise, nanovaccines designed to promote sustained anti-tumor adaptive immune responses [24] may help to overcome limitations of conventional vaccination strategies in the elderly.

This review aims to summarize knowledge on alterations of distinct immune cell types, both in terms of the composition of subpopulations and their functional state and the underlying dysregulated signaling pathways and effector molecules. Next, drugs with reported anti-aging activity on the cellular level and their mechanisms of action will be presented. We will discuss which NC systems as developed primarily for tumor therapy could be employed for directed delivery of anti-aging drugs as well as nanovaccines to improve vaccination success in the elderly.

## 2. Immunosenescence on the Cellular Level

**Figure 1:**
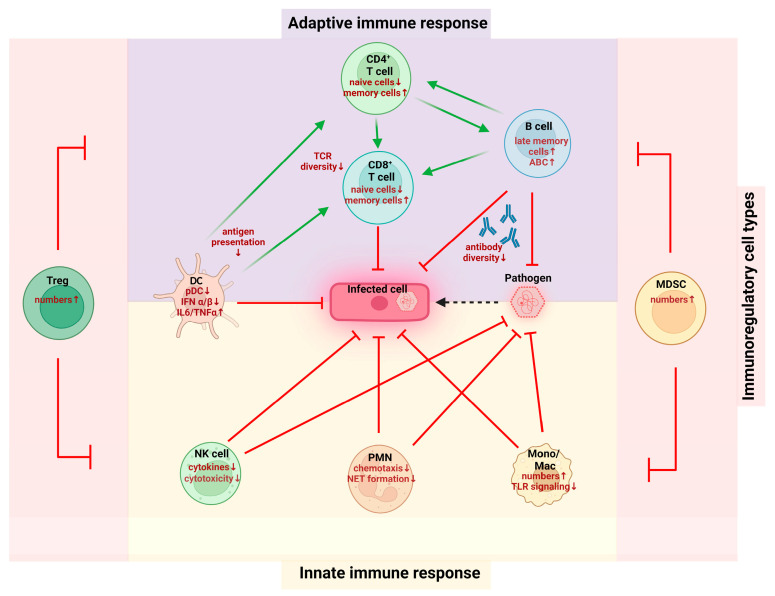
Age-dependent changes in the immune system on the cellular level. Alterations in the number and functions of cell types that belong to the adaptive (**upper part**) and innate (**lower part**) immune system as required to eradicate pathogens and kill infected cells are indicated. The activity of both cell types is inhibited by immunoregulatory cell types that expand upon aging (see list of abbreviations).

The immune system is composed of two separate, but interrelated, parts: an innate arm, comprising the cellular level of monocytes, macrophages, natural killer (NK) cells, and dendritic cells (DCs), and an adaptive arm, namely T cells and B cells [25]. So far, most research on immunosenescence has focused on differentiated immune cells, and major age-dependent alterations are depicted in Figure 1.

However, several studies also suggested that hematopoietic stem cells (HSCs) may undergo changes throughout aging as well [26,27]. In this regard, immunosenescent HSCs are characterized by a decreased clonal diversity [28] and an imbalanced myeloid/lymphoid ratio, driven by the upregulation of genes that are related to myeloid line commitment [29].

### 2.1. Innate Immune Cells

Innate immune cells play a crucial role in conferring inflammaging [30,31]. One reason could be immune challenges throughout life [32]. In addition, damaged macromolecules, organelles, and cell debris can serve as damage-associated molecular patterns (DAMPs) to promote innate immunity especially by activation of the nuclear factor κ-light-chain-enhancer of activated B cells (NF-κB) pathway and by triggering the canonical nucleotide-binding oligomerization domain (NOD)-like receptor family pyrin domain-containing 3 (NLRP3) inflammasome [33]. In this regard it is noteworthy that senescent innate [34] and adaptive [35,36] immune cells are characterized by impaired autophagy, i.e., the lysosome-dependent degradation and recycling of cellular components [37]. Consequently, the overall increase in DAMPs may strongly contribute to inflammaging [38].

#### 2.1.1. Myeloid Cell Types

##### Monocytes/Macrophages

Monocytes constitute motile precursors of tissue-resident macrophages but exert similar functions, such as pathogen recognition, cytokine secretion, and antigen presentation [34]. However, so far most studies have focused rather on immunosenescent aspects of macrophages than monocytes. Macrophages are specialized phagocytic cells that are involved in recognition, phagocytosis, and degradation of pathogens and cell debris. In combination with neutrophil polymorphonuclear granulocytes (PMNs), macrophages constitute the first line of defense to infections. Moreover, activated macrophages also act as APCs [39,40]. Differentiated macrophages are generally divided into classically activated, pro-inflammatory “M1-like” and alternatively activated, anti-inflammatory “M2-like” macrophages, although the existence of respective subpopulations has been recognized [41].

Hearps and coworkers reported on alterations in the composition of monocytes in the elderly with a decrease in the main population of cluster of differentiation (CD)14^+^CD16^−^ monocytes and concomitantly an increase in so-called intermediate CD14^+^CD16^+^ and non-classical CD14^dim^CD16^+^ monocytes [42]. In a geriatric mouse model, increased numbers of macrophages were observed in the bone marrow and in the spleen [43]. However, in the bone marrow of old patients no significant changes were noted [44]. Further, in mice numbers of hepatic macrophages accumulated with age [45].

Several studies reported reduced expression of Toll-like receptors (TLRs) in monocytes [42] and macrophages of the elderly, thereby limiting their responsiveness towards DAMPs [46]. Further, splenic macrophages derived from aged mice showed impaired activation of NF-κB and p38 mitogen-activated protein kinase (MAPK) signaling in response to TLR-4 activation [47]. Similarly, human blood monocytes of the elderly showed attenuated expression of TLR-1 and impaired activation of extracellular signal-regulated kinase (ERK)1/2 when applying a TLR-1/2 agonist [48].

In this line, microarray analysis of lipopolysaccharide (LPS)-stimulated murine M1-like macrophages revealed lower induction of genes encoding pro-inflammatory cytokines/chemokines in the case of cells derived from aged mice [49]. Likewise, macrophages of the elderly were characterized by an attenuated production of tumor necrosis factor (TNF)-α, interleukin (IL)-6, and IL-1β upon *S. pneumoniae* infection and an impaired capacity to kill the pathogen compared to macrophages of younger adults [50]. Further, activated macrophages of old mice presented with decreased major histocompatibility complex (MHC)II expression leading to impaired antigen presentation to T cells [51]. Consequently, old mice presented with a higher susceptibility to infection [51,52]. Interestingly, aging also resulted in impaired activity of M2-like macrophages in wound healing [53].

##### Microglia

The central nervous system (CNS) is also affected by immunosenescence. Microglia, the brain-resident macrophage population, constitutes the main immune cell type of the CNS and accounts for roughly 10% of all cells in the brain [54]. Aside from immune surveillance, the microglia are important for maintaining brain homeostasis [55] and undergo various changes throughout aging, such as elevated secretion of pro-inflammatory cytokines [56] and impaired motility and phagocytosis [57]. These processes drive neuroinflammation, which is a hallmark for many neurodegenerative diseases like Alzheimer’s disease (AD) and Parkinson’s disease (PD) [58].

##### PMNs

PMNs are the most abundant leukocyte in the blood and constitute the first line of defense against bacterial, fungal, and yeast pathogens at infected sites [59,60]. Neutrophils have a rather short lifespan under homeostatic conditions but persist much longer upon stimulation, e.g., by pathogens [61].

Aging was not found to alter the numbers of circulating PMNs in healthy and hospitalized patients [62,63]. Also, their adhesion to endothelium at inflamed sites and tissue immigration was not affected by age [64]. However, human PMNs isolated from aged people were characterized by decreased chemotaxis towards granulocyte–macrophage colony-stimulating factor (GM-CSF), N-formylmethionyl-leucyl-phenylalanine (fMLP), and LPS [65], and similar findings were obtained for PMNs derived from aged mice [66]. Further, in aged mice reverse transendothelial migration of PMNs to re-enter the circulation occurred more frequently, which was mediated by increased production of chemokine C-X-C motif ligand (CXCL)1 by tissue-resident mast cells [67]. On a functional level, human PMNs derived from the elderly exerted lower phagocytic activity [68]. Both defects in migration and phagocytosis may be attributed at least in part to dysregulated expression of required effector proteins.

In this regard the β2 integrins CD11a/CD18 (lymphocyte function-associated antigen 1, LFA-1) and CD11b/CD18 (macrophage-1 antigen, MAC-1), which commonly interact with intercellular adhesion molecule 1 to facilitate leukocyte rolling as a prerequisite for transendothelial migration, play an important role [69]. Moreover, MAC-1 recognizes complement C3-opsonized pathogens and immune complexes and mediates their phagocytic uptake [70]. In addition, MAC-1 constitutes a coreceptor of Fc receptors, which confer internalization of antibody-opsonized pathogens and immune complexes [71,72]. Whereas Butcher and coworkers observed no age-dependent alterations of LFA-1 and MAC-1 on PMNs [68], Gasparato et al. reported on attenuated MAC-1 expression in PMNs derived from the elderly [73]. In the same study, PMNs derived from aged people showed increased expression of FcγRIIa (CD32) but lower surface levels of FcγRIII (CD16). The latter was also observed by Butcher and coworkers [68]. Furthermore, CD32 expression was refractory towards stimulation-induced upregulation in PMNs of the elderly, whereas MAC-1 was strongly upregulated [73]. In contrast to humans, in the same study murine PMNs were found to express and upregulate Fcγ receptors to a similar extent, irrespective of mouse age. Further, murine PMNs of old mice expressed MAC-1 to a higher extent under basal conditions but were refractory towards stimulation which resulted in MAC-1 upregulation in PMNs of young mice. This age-dependent defect was not observed in human PMNs. However, in human monocytes [74] and T cells [75] LFA-1 expression increased with age.

Besides attenuated chemotactic and phagocytic activity, murine PMNs of aged mice were also demonstrated to release fewer neutrophil extracellular traps (NETs), comprising chromatin and bactericidal mediators derived from the cytoplasm and granules [76], in response to pathogens, and to produce attenuated amounts of CXCL2 [77]. CXCL2 serves to attract PMNs, thereby promoting a positive feedback loop [78]. The impaired response of PMNs derived from the elderly towards GM-CSF has been attributed to the elevated activity of different inhibitory proteins like Src homology domain-containing protein tyrosine phosphatase-1 (SHP-1) and suppressors of cytokine signaling (SOCSs) [79]. As mentioned above, tissue repair is negatively affected by advanced age [80]. In association with defective tissue regeneration, the abundance of CD11b^+^Ly6C^int^Ly6G^+^ PMNs significantly decreased with age [81].

##### MDSCs

Myeloid-derived suppressor cells (MDSCs) are a heterogeneous group of myeloid cells, which expand in the course of infections and tumor progression [82]. In cancer, MDSCs in combination with regulatory T cells (Treg) contribute to tumor immune evasion by inhibiting the activity of APCs and T effector cells by various mechanisms [83]. In aged mice, elevated numbers of MDSCs have been observed in bone marrow, spleen, lymph nodes, and blood [84]. Also, in elderly humans MDSC numbers were increased [85]. Further, in aged mice MDSCs were shown to exert an elevated T cell inhibitory function, attributed in part to elevated activities of arginase-1 [86,87] and inducible nitric oxide synthase (iNOS) [87]. The latter was considered a consequence of age-dependently impaired PI3K signaling, which resulted in enhanced release of IFN-γ triggering both iNOS expression and activity.

#### 2.1.2. DCs

Activated DCs are the most potent APCs due to their capacity to induce primary T cell responses [88]. DCs comprise several subpopulations, including conventional (c)DCs and plasmacytoid (p)DCs, which differ in their main functions [89]. Whereas cDCs produce IL-12 and activate helper T cells (Th)1, pDCs express surface markers of both myeloid and lymphoid origin and secrete interferon (IFN)α/β in response to (viral) infection [90,91].

While the total number of cDCs was reported as largely unaltered by age, the abundance of pDCs was shown to decline with age [92,93,94,95]. DCs in aged humans and mice were found to contribute to inflammaging by producing IL-6 and TNF-α even in an unstimulated state [96]. However, DCs from aged individuals were characterized by an increased production of the pro-inflammatory cytokines TNF-α, CXCL-10, and IL-6 in response to LPS [97] and *Chlamydophila pneumonia* [98], whereas a lower level of the anti-inflammatory cytokine IL-10 was noted in DCs obtained from the elderly [97]. In elderly humans derived pDCs showed lowered IFN-I secretion upon infection [99,100] due to impaired interferon regulatory factor (IRF)-7 activation [100] and lower IRF-8 induction [101]. Similarly, monocyte-derived DCs derived from the elderly were reported to generate lower amounts of IFN-I and IFN-III in response to stimulation, associated with preferential association of IFN promoters with inhibitory histone H3K9me3 and lower association with activating H3K4me3 [102]. For total DC populations attenuated endocytic capacity as well as antigen presentation after stimulation were reported [103]. In agreement, DCs derived from old mice showed impaired CD4^+^ [94] and CD8^+^ [104] T cell stimulatory capacity.

#### 2.1.3. NK Cells

NK cells represent approximately 15% of peripheral blood lymphocytes [105], which recognize malignant cells due to dysregulated MHCI expression and exert direct cytotoxic effects [106,107]. The number of CD56^dim^ NK cells was found elevated in older humans, but their cytotoxic capacity on a single-cell level was attenuated [108]. At the same time the number of CD56^hi^ NK cells, which exert immune modulatory functions, was lowered in an age-dependent manner [109]. Similarly, NK cells derived from aged humans produced fewer cytokines (IL-2, IFN-γ, TNF-α and IL-12) [110,111] and chemokines (C-C motif chemokine ligand (CCL)3, CCL5, IL-8) [112] after stimulation.

### 2.2. Adaptive Immune Cells

The adaptive immune system consists of peptide antigen-specifically activated T cells of which CD8^+^ cytotoxic T lymphocytes (CTL) exert direct cytotoxic activity on pathogen-infected and malignant cells [113] and CD4^+^ Th confer coactivation of CD8^+^ T cells and B cells [114]. Activated protein antigen-specific B cells release antigen-specific antibodies that may opsonize pathogens and pathogen-infected cells [115]. This may on the one hand trigger the classical complement activation pathway yielding complement-mediated killing [116] and, on the other hand, may cause phagocytic uptake via engagement of Fc receptors on myeloid cells [117]. In the past, age-related changes in the adaptive immune system were considered the primary cause of immunosenescence [118].

#### 2.2.1. T Cells

One of the most significant changes in the immune system throughout aging is the loss of thymic mass due to thymic involution, which results in diminished numbers of naïve CD4^+^ and CD8^+^ T cells [119,120]. In accordance with encounters with numerous pathogens throughout life, the number of memory T cells is increased in aged individuals [121]. In accordance, their T cell population displays an overall reduced clonal diversity of T cell receptors [122]. In addition, T cells of aged individuals are characterized by attenuated T cell receptor (TCR)/CD28 signaling efficacy as a consequence of increased membrane cholesterol levels that cause attenuated membrane fluidity [123] and show impaired responsiveness towards IFN-I signaling [124]. Whereas the total number of CD4^+^ T cells in blood was found unaffected by aging, CD8^+^ numbers were diminished [125,126]. The frequency of CD4^+^ T cells with an exhausted immunophenotype was shown to be increased in old mice [127]. Especially Th1 and Th17 were reported to exert diminished activity in older people [128]. In addition, a higher frequency of CD8^+^ T cells in the elderly displayed a memory phenotype at the expense of naïve T cells and showed a senescent phenotype [129,130]. In addition, the number of Tregs was found to be increased in aged individuals, but conflicting data suggested that these may exert either an elevated [127,131] or reduced [132] immunosuppressive function. In the latter study, Tregs derived from old mice were characterized by attenuated expression of DDB1- and CUL4-associated factor 1, which was required to inhibit the production of pro-inflammatory cytokines and to retain T cell inhibitory activity.

#### 2.2.2. B Cells

B cells act, on the one hand, as APCs [133] and, on the other hand, produce antigen protein-specific antibodies [115]. It was demonstrated that B cell development in the bone marrow was disturbed by aging as the overall number of common lymphoid progenitors and the proliferative capacity of derived pro- and pre-B cells were reduced, associated with higher levels of apoptosis [134]. This affected classical antibody-generating B2 cells, whereas B1 cells, that constitutively generate antigen-unspecific immunoglobulin (Ig)M, were unaffected [135].

Numbers of age-associated B cells (ABCs) that display a pro-inflammatory state were found to increase with age [136], and de novo antibody production of B cells decreased during aging [137]. ABCs contribute to immunosenescence by attenuating the generation of B cells [138]. B cell responses may also be hampered by attenuated activity of Th [139] and follicular helper T cells (Tfhs) [130].

Interestingly, drastically increased frequencies of ABCs that largely produce autoantibodies are also a hallmark in patients suffering from autoimmune diseases, for example, arthritis and systemic lupus erythematosus [140]. However, ABCs that frequently expand in the course of infection play a protective role upon reinfection [141].

## 3. Role of Impaired DNA Stability and Altered Gene Expression for Immunosenescence

**Figure 2:**
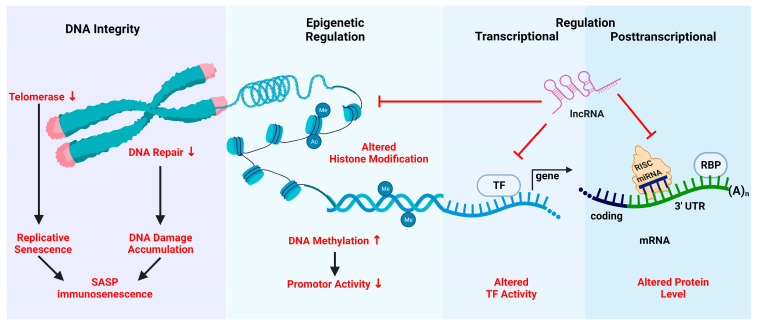
Immunosenescence as a consequence of attenuated genomic stability and altered gene expression. On the genomic level aging is associated with telomere attrition and thereby replicative senescence as well as attenuated repair of DNA damage. Both result in immunosenescence. Further, age-dependent alterations on the epigenetic level, comprising histone modifications and the methylation of CpG residues located predominantly in gene promoter regions, affect the accessibility for TF. Epigenetic changes as well as differential TF expression/activity affect gene expression on a transcriptional level. Furthermore, gene expression is modulated on a posttranscriptional level by lncRNA species that affect epigenetic regulation, TF activity and mRNA stability, differentially expressed miRNA, and RBP (see list of abbreviations).

Aging can be described as the gradual functional decline affecting most living organisms and has been a subject of research since the 1980s [142,143]. Aging-associated major cellular and molecular changes include genomic instability, epigenetic alterations, mitochondrial dysfunctions, and cellular senescence caused mainly by telomere attrition [144]. Since immune cells are also affected by aging, these events drive immunosenescence. In the following, age-associated effects on the different layers of gene regulation are discussed and summarized schematically, as shown in Figure 2.

Uyar and coworkers compared multi-tissue single-cell RNA-sequencing data from previously published studies and found several genes associated with senescence and inflammaging to be upregulated in old mice [145]. For example, in the *Tabula Muris Senis* study, IL-1β, a cytokine involved in inflammaging [146], was found to be expressed by nearly all Kupffer cells (KCs) in the liver of old mice, whereas in young mice less than 50% of KCs expressed IL-1β [147]. Expression of IL-1β was also upregulated in various other cell types in the liver, including B cells, hepatic stellate cells, liver sinusoidal endothelial cells, myeloid cells, and pDCs. In the following, age-dependent changes on the various genomic and gene regulation levels are briefly discussed.

### 3.1. Telomeres

Telomeres are regions of repetitive guanine-rich nucleotide sequences at the ends of linear chromosomes [144] and are associated with shelterin proteins, which protect the exposed ends of telomeres from the DNA damage machinery [148,149]. Telomeres are important for genomic stability by overcoming the so-called “end replication problem” [150]. In brief, DNA polymerases are unidirectional enzymes and can only synthesize DNA in the 5′–3′ direction. Since the lagging strand is oriented 3′–5′, its replication is discontinuous and requires repeated synthesis of RNA primers binding further 5′ sequences. This leads to loss of DNA at the 5′ end of the lagging strand after removal of the primers after replication. Therefore, telomeres become shorter with every replication cycle and eventually reach a critical length [151]. Then, the DNA damage response is activated, leading ultimately to p53-induced apoptosis or cellular senescence, i.e., irreversible cell cycle arrest [152]. The relevance of telomeres for aging is highlighted by the fact that several premature aging disorders are characterized by shortened telomere length or accelerated telomere attrition. In general, telomere-related senescence is a major cause of SASP [153].

Telomerase is an enzyme that counteracts telomere shortening by adding DNA to telomeres via reverse transcription. It consists of the catalytic protein component telomerase reverse transcriptase (TERT) and a telomerase RNA component (TERC). The enzymatic activity of telomerase is inhibited in most somatic cells but is maintained in proliferating tissue, germ cells, and activated immune cells [154]. A number of transcription factors (TFs) have been reported to influence human (h)TERT promoter activity. For instance, signal transducer and activator of transcription (STAT)3 and STAT5, cellular myelocytomatosis (c-myc), and hypoxia-induced factor (HIF)-1 upregulate hTERT expression, whereas p53, mainly activated by DNA damage, and E2 transcription factor 1 inhibit its transcription [155,156]. Vice versa, the age-associated decrease in telomere length was associated with attenuated STAT5a activity as delineated in macrophages [157].

Blood-derived human HSCs were demonstrated to display diminished telomerase activity, accompanied by enhanced oxidative stress and pyroptosis [158], i.e., inflammasome-induced cell death [159]. In activated lymphocytes telomerase expression is increased mainly by translocation of NF-κB to the nucleus to prevent senescence during clonal expansion [154]. Additionally, posttranslational modifications, such as phosphorylation, also influence hTERT activity and its cellular localization.

Elevated telomerase activity cannot fully prevent telomere attrition and cellular senescence in T cells, which is fueled by recurring infections and immune cell activation throughout life [153,160]. In fact, senescent T cells are characterized by low expression of telomerase, lack CD28 and CD27 surface marker expression, and show increased release of the pro-inflammatory cytokines TNF-**α** and IL-1β as typical SASP components and thereby contribute to inflammaging [161]. Interestingly, chronic infection with a large variety of viruses has been demonstrated to result in telomere attrition and thereby replicative senescence of virus-specific CD8^+^ T cells [162].

Unlike T cells, B cells do not show telomere erosion upon rapid proliferation, and telomere length in memory B cells is comparable to naïve B cells [163]. This is mainly due to stable expression of telomerase in B cells. However, B cells also show progressive telomere shortening with age, which is caused by a multitude of factors, such as obesity [164], oxidative stress [165], viral infections [166], and inflammation [153].

Of note, murine Tregs were reported to inhibit telomerase activity in various types of lymphocytes in a cell contact-independent manner via transfer of endonuclease G, thereby causing telomere-associated senescence and consequently apoptosis [167,168]. In light of the elevated number and activity of Tregs in the elderly [169] further studies should address whether this mechanism contributes to immunosenescence. Further, PMNs were reported to cause telomere shortening in cocultured fibroblasts in a cell contact- and ROS-dependent manner, thereby inducing replicative senescence [170]. Interestingly, hepatocytes of aged mice were found to upregulate expression of PMN-recruiting chemokines, associated with elevated numbers of senescent liver cells. In a model of acute liver injury, inflammation-induced cellular senescence was attenuated upon depletion of PMNs and inhibition of ROS activity.

### 3.2. DNA Repair

Genomic integrity is under constant attack by genotoxic agents, such as UV and ionizing radiation, reactive oxygen species (ROS), and chemicals that cause DNA damage [171]. Most DNA damage is repaired by the DNA damage response (DDR) system, but DNA damage accumulates during aging, leading ultimately to genomic instability since not all DNA lesions are repaired correctly [172]. Persistent DNA damage has been found to be a causal factor for the aging process [173]. The DDR is mainly activated by the kinases ataxia telangiectasia mutated and ataxia telangiectasia and Rad3-related protein, which lead to downstream activation of the tumor suppressor p53 [174]. p53 then either initiates DNA repair, apoptosis, cell cycle arrest, or senescence [175]. Yousefzadeh and coworkers showed that knocking out the DNA repairing protein excision repair cross-complementation group increased DNA damage, leading to an earlier onset of immunosenescence and accelerated systemic aging [176]. Grasselli et al. found significantly increased levels of damaged DNA in circulating HSCs of elderly frail adults, compared to non-frail elderly, indicating that DNA damage in these cells drives frailty, an age-related syndrome, characterized by increased vulnerability and higher mortality [177]. Besides DNA damage, cytoplasmic chromatin fragments [178,179] and mitochondrial DNA [180] as released by senescent cells via activation of the cyclic GMP-AMP synthase-stimulator of interferon genes pathway may also spread SASP. Besides, senescence-dependent activation of the retrotransposable element long interspersed nuclear element 1 induced IFN-I as an anti-viral response and thereby contributed to inflammaging [181].

### 3.3. Gene Regulation

#### 3.3.1. Epigenetic Regulation

The term “epigenetic” describes modifications of the DNA which are not associated with alterations of the DNA sequence itself [182]. Epigenetic mechanisms include DNA methylation, histone modifications, and chromatin remodeling. The chromatin structure can be divided broadly into euchromatin, which is transcriptionally active, and heterochromatin, which contains many CpG methylated areas and shows low transcriptional activity. Aging has been associated with loss of heterochromatin and derepression of silenced genes by epigenetic modifications of the relevant loci [183]. For example, Sun et al. analyzed the transcriptome, DNA methylome, and histone modifications of young and aged murine HSCs and found increased methylation (and therefore silencing) of genes associated with differentiation and decreased methylation of genes known to be important for self-renewal [184]. Based on tissue-wide analysis, age-correlating DNA methylation patterns have been elucidated and termed “epigenetic clocks” [185]. An assay identifying transposase-accessible chromatin by sequencing (ATAC-seq) of human peripheral blood mononuclear cells (PBMCs) revealed a reduced accessibility of regions important for T cell signaling and simultaneous higher accessibility of quiescent and repressed sites. This aging signature of PBMCs was most pronounced in CD8^+^ T cells which showed age-related silencing of the IL-7 receptor gene and loss of NF-κB and STAT binding to the IL-7 receptor gene promotor [186]. Other age-related changes in various immune cells have been reviewed in [183].

#### 3.3.2. Transcription Factors

As outlined in the following, age-dependently dysregulated expression and activity of transcription factors (TFs) contribute to immunosenescence by affecting both hematopoiesis as well as the activation and polarization of immune cells.

##### HSCs

FOXO3 was shown to play a crucial role in HSC homeostasis by enhancing the expression of genes required for repair of oxidative DNA damage [187] and promoting mitochondrial metabolism [188] and autophagy [189]. In the elderly, FOXO3 was demonstrated to be downregulated by age-induced miRNA species (see below). Further, p53-induced phosphatase Wiskott–Aldrich syndrome protein-interacting protein (WIP)1 [190] was reported as required for HSC repopulation efficacy [191]. Consequently, impaired HSC activity in the elderly may be in part a consequence of age-dependent downregulation of both p53 and WIP1 [192].

##### Adaptive Immune Cells

In human T cells and B cells an age-associated impaired expression of BTB domain and CNC homolog (BACH)2 has been observed [193]. BACH2 was shown to promote the differentiation of CD4^+^ T cells towards Tregs [194] and to delay the differentiation of B cells towards plasma cells to enable class switch recombination and somatic hypermutation of immunoglobulins [195]. BACH2 downregulation was accompanied by an increase in BACH2-repressed PR domain-containing 1, with ZNF domain (PRDM1), demonstrated to promote both T cell and B cell differentiation [196]. The age-dependent shift in expression of both TFs may contribute to the overall attenuated numbers of naïve lymphocytes in the elderly. Autophagy describes the lysosomal degradation of cellular macromolecules and is downregulated in an age-dependent manner [197]. The endogenous polyamine metabolite spermidine, which drives expression of the autophagy-inducing transcription factor EB [198], was found downregulated in T cells [199] and B cells [200] of the elderly. In vitro application of spermidine largely restored functional activity of both adaptive immune cell types.

##### T Cells

T cell factor (TCF)1 activity is necessary for T cell development as well as T cell differentiation [201]. TCF1 mRNA expression was reduced in the elderly due to hypermethylation of the gene promoter [202] and attenuated expression of forkhead box protein (FOX)O1 that confers expression of TCF1 [203]. On a posttranscriptional level, TCF1 was inhibited by elevated levels of microRNA (miRNA)-21 [204]. FOXO1 was shown to be downregulated by sustained PI3K and mTOR activity in activated T cell populations of the elderly [205]. Of note, TCF1 also conferred expression of miR-181a, which in turn positively regulates TCR activation by inhibiting the expression of TCR-attenuating phosphatases [206]. Therefore, attenuated TCF1/miR-181a may explain in part the age-dependently reduced T cell activity. In general, exhausted T cells that also arise in the course of chronic infection [7] and cancer [207] are characterized by TCF1 deficiency [208].

Besides TCF1, B cell lymphoma (BCL)-6 expression was also reduced in activated T cells of aging donors [204]. Both TFs are required for the differentiation of activated CD4^+^ T cells towards Tfhs [209]. In addition, FOXO1 is necessary for CD8^+^ T memory cell differentiation [210]. T cells of the elderly were characterized by elevated expression of the TF PRDM1 and of the runt-related TF (RUNX)3 [193,211]. Of these, PRDM1 was reported to promote Tregs [212] and to inhibit Tfh differentiation [213], whereas RUNX3 promoted Th1 in favor of Th2 differentiation [214,215]. Hu and coworkers reported on preferential Th9 polarization of activated CD4^+^ T cells of the elderly due to enhanced responsiveness towards TGF-β via increased expression of TGF-β receptor 3 elevating expression of spleen focus forming virus pro-viral integration oncogene (SPI)1 [216]. SPI1 is required to confer Th9 polarization [217,218]. In the same study the basic leucine zipper TF and ATF-like [219] and IRF4 [220] previously demonstrated to promote Th9 differentiation were also found upregulated in CD4^+^ T cells obtained from the elderly. Most recently, Zhang et al. demonstrated that activated CD4^+^ T cells derived from the elderly displayed attenuated expression of HELIOS as compared to CD4^+^ T cells obtained from younger donors and concomitantly elevated phosphorylation of STAT5 [221]. Activated STAT5 conferred vast epigenetic changes and consequently changes in the gene expression profile, promoting the differentiation towards inflammatory effector cells.

##### B Cells

Piskor and coworkers demonstrated that inactivation of Myc-interacting zinc finger protein 1 (Miz-1) mimicked features of B cell immunosenescence in B cells downstream of the pro-B cell state [222]. Further, BCL-6 interacted with Miz-1 to inhibit expression of cyclin-dependent kinase inhibitor, thereby promoting germinal center formation [223], which is strongly affected in the elderly [224]. However, it needs to be shown whether Miz-1 activity is altered in an age-dependent manner. Common lymphoid progenitors of aged mice were found to express lower levels of early B cell factor and paired box (PAX)5 [225]. Forced expression of constitutively active STAT5 restored expression levels of both TFs and rescued age-dependent defects in B cell generation. Expression levels of E47 and its target activation-induced cytidine deaminase, which are both required for Ig class switching and somatic hypermutation of Ig genes [226], were attenuated in B cells of the elderly [227]. B cells derived from adults were reported to contain higher levels of inhibitor of binding or differentiation 2 that by inhibiting its target E47 interferes with immunoglobulin gene rearrangements in B cells [228]. Therefore, B cells in the elderly are less able to optimize antibody affinity to pathogenic proteins [229]. T-box expressed in T cells has been suggested as crucial for the differentiation of murine [230] and human [231] ABCs like those induced by Epstein–Barr virus (EBV) [232] and in systemic lupus erythematosus patients [233]. In the latter study IRF5 was identified as an ABC-promoting factor. However, it is unclear yet whether IRF5 is also involved in age-associated ABC differentiation. Additionally, ABCs obtained from older individuals displayed lower paired box (PAX)5 levels as compared to the ABC population of younger donors [234]. Further, levels of PAX5 were also diminished in ABCs obtained from older individuals that generated only poor antibody amounts 2–4 months after influence vaccination and therefore were considered non-responders as compared to ABCs obtained from elderly vaccination responders.

##### Inflammaging

Early on, the NF-κB family members p52 and p65 were recognized to display elevated activity in immune cells obtained from aged mice [235] and to constitute the main TFs driving inflammaging and SASP in immune cells as induced, for example, by DNA damage [236] and as a consequence of activated MAPK p38 signaling [237]. The activities of both MAPK p38 [238] and NF-κB [239] are negatively regulated by WIP1. Age-dependent downregulation of the WIP1 regulator p53 and WIP1 thereby may contribute to promotion of SASP [192]. Further, in different non-immune senescent cells Janus kinase activity promoted a SASP phenotype [240]. More recently, both innate and adaptive immune cells of mice were demonstrated to display enhanced expression of several activator protein-1 TF family members that may contribute to SASP as exemplified by Jun driving IL-6 production in myeloid cells of aged mice [241]. In senescent cells the histone H3 lysine (K)-specific demethylase 4 (KDM4) was identified as upregulated and to confer expression of SASP-associated factors [242]. In human monocytes KDM4 was identified as an important positive regulator of cytokine responses induced by repetitive stimulation [243]. It remains to be shown whether KDM4 also plays a role in inflammaging in immune cells.

#### 3.3.3. Non-Coding RNA

##### Long Non-Coding (lnc)RNA

lncRNA (>200 nucleotides) acts on several levels to regulate gene expression, including epigenetic modulators like histone modifiers as exemplified by lncRNA-AW112010, which promoted CD4^+^ T cell activation by engaging the histone demethylase lysine demethylase 5A [244]. By this, histone H3K4 methylation of the IL-10 gene locus was reduced and IL-10 production attenuated. Further, several lncRNAs were reported to modulate the activity of DNA methyltransferase, for example, in polarizing Th populations [245]. Concerning the CD8^+^ T cell compartment, the lncRNA-NRON was found downregulated in senescent CMV_pp65_CD8^+^ CD28^-^ T cells of the elderly, whereas its target gene nuclear factor of activated T cells was concomitantly upregulated [246].

lncRNA may also control the activity of TFs, for example, lncRNA-Gm9866, which promoted M2 macrophage polarization by inhibiting NF-κB activity [247], and act on a posttranscriptional level by various mechanisms. For example, in activated B cells lncRNA-XLOC_098131 acted as a decoy substrate for miR-548, resulting in elevated stability of the Fos proto-oncogene, activator protein-1 transcription factor subunit encoding mRNA, and consequently elevated B cell survival and differentiation towards plasma cells [248]. In macrophages, stimulation-induced lncRNA-FIRRE interacted with the RNA-binding protein heterogeneous nuclear ribonucleoproteins U stabilizing vascular cell adhesion molecule 1 and IL-12p40 mRNA [249].

Several lncRNAs have been shown to regulate cellular senescence, for example, lncRNA-SENEBLOC by inhibiting expression of the senescence effector p21 [250]. However, further studies are required to elucidate the contribution of lncRNAs that regulate signaling pathways like NF-κB and of pro-inflammatory cytokines as SASP components for immunosenescence.

##### miRNA

In contrast to lncRNA, miRNA (~22 nucleotides) regulates gene expression exclusively on a posttranscriptional level [251]. miRNAs are embedded in the so-called RNA-induced silencing complex and bind sequence-complementary stretches in mRNA [252]. In case of several mismatches, target mRNA translation is inhibited by various mechanisms, including interference with binding of ribosomal factors, while near perfect binding of miRNA affects mRNA half-life. As outlined above, FOXO3 plays an important role in HSC maintenance [253]. Disturbed hematopoiesis in the elderly may be in part a consequence of age-dependently upregulated expression of members of the miR-132/212 cluster in HSCs, which in turn inhibit FOXO3 expression [254]. In macrophages derived from aged mice stimulation elevated expression of induced cyclooxygenase (COX-)2 mRNA to a much higher extent as observed in macrophages obtained from young mice due to impaired expression of the COX-2 mRNA inhibiting miR-26b and miR-101b in the former group [255]. The authors showed that expression of these miRNA species was attenuated in macrophages of old mice on an epigenetic level by histone deacetylation. As another example, the regulation of stimulation-induced miR-146a was found dysregulated in aging mice [256]. Macrophages of old mice displayed about 6-fold higher expression of miR-146a in unstimulated macrophages as compared to the expression level of cells of young mice. Further, whereas LPS stimulation increased expression of miR-146a by 12-fold in macrophages derived from young mice, no upregulation was observed for cells obtained from old mice.

Further, Xu et al. observed an age-correlating decrease in miR-17, miR-92a, and miR-181a expression in human PBMCs [122], which was in line with previous observations [257,258] and correlated with decreased TCR sensitivity [259] and reduced numbers of naïve CD8^+^ T cells [6]. Activated CD4^+^ T cells of elderly donors showed much stronger expression of miR-21 than T cells obtained from younger donors, causing attenuated expression of various TFs (e.g., TCF1 and BCL6) and activation of ERK and mTOR signaling, all contributing to the differentiation towards short-lived effector cells at the expense of memory precursors [260].

#### 3.3.4. RNA-Binding Proteins (RBPs)

RBPs also control gene expression on a posttranscriptional level [261]. To this end, RBPs engage cognate recognition sites that are located most often within the 3′-untranslated region of their target mRNA and may modulate mRNA stability and translational efficacy, respectively. Several RBPs have been implicated in the regulation of cellular senescence, for example, pumilio (Pum)2 which was found induced in aging mice [262]. Pum2 was identified as a translational repressor of mitochondrial fission factor, resulting in attenuated mitophagy. Likewise, human antigen (Hu)R is among several RBPs which have been reported as upregulated in humans in an age-dependent manner [263]. Of these, HuR has been shown to prevent mitochondrial ROS production by inhibiting telomeric repeat-binding factor 1-interacting nuclear factor 2 mRNA stability and translation [264] to promote telomerase expression via association with TERC [265] and to enhance expression of the cell lifespan regulator SIRT1 [266,267] and of the autophagy regulator autophagy-related 7 [268] via mRNA stabilization. In addition, HuR has been reported to inhibit the production of pro-inflammatory cytokines in cellular senescence models [269]. Therefore, HuR may serve to compensate age-related cellular senescence.

In contrast to the aforementioned RBPs, tristetraprolin (TTP) was found downregulated in the elderly [263]. Kwack and coworkers demonstrated that TTP expression was also diminished in myeloid cell types of old mice, which was associated with an increase in mMDSCs in spleen and of gMDSCs in bone marrow [270]. The potential role of TTP for age-related alterations in myelopoiesis was assessed in young TTP-deficient mice. These presented with higher numbers of both MDSC populations in secondary lymphoid organs, which were attributed to higher frequencies of the respective progenitor cells, and a higher expression of C-C chemokine receptor type (CCR)2 on myeloid cells as well as elevated concentrations of CCL2 in serum. The authors concluded that in old mice TTP limited the number of myeloid progenitor cells and enhanced CCL2/CCR2-mediated recruitment of derived MDSCs into the periphery. In contrast to the finding of attenuated TTP levels in myeloid cells, B cells derived from the elderly were shown to contain an overall higher amount of active hypophosphorylated and thereby active TTP [271]. Hyperactive TTP facilitated degradation of mRNA encoding the TF E47 [229], which is required for immunoglobulin class switch [272]. The RBP zinc finger protein 36, C3H type-like (ZFP36L)1 belongs to the same RBP family as TTP and accordingly inhibits the half-life of its mRNA targets [273]. In model cell lines, ZFP36L1 was identified as a major inhibitor of SASP components [274]. In senescent cell lines, mTOR was found to enhance expression of MAPK-activated protein kinase 2, which in turn phosphorylated and thereby inhibited ZFP36L1 activity, confirming a role of this signaling cascade in inflammaging.

Nuclear factor of activated T cells 90 KDa (NF90) was found to act both as an RBP and in combination with NF45 as a transcriptional regulator [275] and modulator of primary miRNA processing [276]. NF90 has been identified as a repressor of pathogen-induced cytokine production in monocytes including, e.g., TNF-α and CCL2, by inhibiting both mRNA stability and translational efficacy [277] and as a transcriptional repressor of stimulation-induced human DC maturation by affecting MHCII and costimulator (CD40, CD86) expression [278]. NF90 was found to be downregulated in senescent fibroblasts which in turn elevated the level of SASP-related cytokines like IL-6 and IL-8 due to enhanced mRNA levels [279]. Further studies are necessary to delineate the potential involvement of NF90 in aging immune cells.

## 4. Dysregulated Signaling Contributes to Immunosenescence

**Figure 3:**
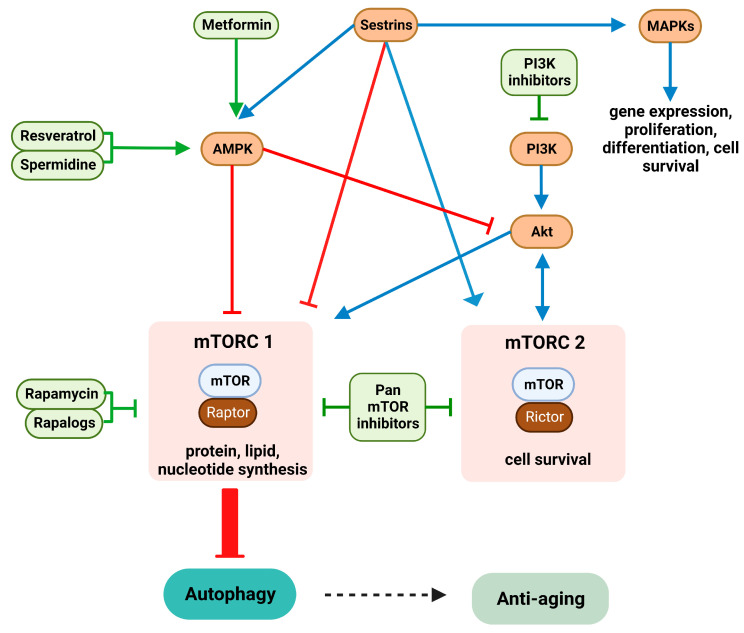
Signaling pathways dysregulated in aging and suitable modifiers. Signaling pathways dysregulated in aging (blue) and drugs (green) shown to restore normal function are shown (see list of abbreviations).

Dysregulation of several signaling pathways is a hallmark of immunosenescent cells (Figure 3). AMP-activated protein kinase (AMPK) acts as a sensor of cellular energy status and regulates metabolic homeostasis and cell survival during stress [280]. Gene expression analysis of PBMCs derived from older individuals after influenza vaccination showed a correlation in the expression of various immunosenescence markers and constituents of the mTOR (see below) and AMPK pathways [281]. AMPK is a Ser/Thr kinase composed of catalytic α- and regulatory β- and γ-subunits, and each of these exists in 2–3 isoforms [282]. AMPK is activated in case of ATP depletion by increased ratios of AMP/ATP and ADP/ATP. Additionally, several upstream kinases can activate AMPK by phosphorylating the α-subunit at Thr172. These include serine/threonine kinase 11 [283], Ca^2+^/calmodulin-dependent protein kinase β [284], and TGF-β-activated kinase 1 [285]. The AMPK pathway has multiple important functions in immune cells [286]. For instance, AMPK activation was demonstrated to inhibit LPS-mediated DC and macrophage activation by attenuating NF-κB activity [287]. Further, AMPK constitutes a key metabolic control point for transition from glycolysis to fatty acid oxidation and oxidative metabolism in activated T cells [288].

PI3K is a family of enzymes involved in several cellular functions including proliferation, differentiation, and survival [289]. Concerning immune cells, PI3K activity was shown to be significantly upregulated in PMNs of old as compared to young donors and inversely correlated with migratory capacity [290]. PI3K inhibition restored migration accuracy of PMNs whereas blockade of SHP-1, an inositol phosphatase that blocks PI3K signaling [291], impaired migration [290].

Both AMPK [292] and PI3K [293] regulate the mTOR pathway. In addition, mTOR signaling is also induced by amino acids [294,295], insulin [296], and Ras homolog enriched in the brain [297]. mTORs are Ser/Thr kinases belonging to the PI3K-related kinase family and control various processes, such as transcription, ribosome formation, translation, cell growth, and autophagy [298]. In mammals, mTOR exists in two isoforms, mTOR1 and mTOR2, which interact with different proteins and are constituents of distinct complexes [299]. mTOR complex 1 (mTORC1) is rapamycin-sensitive and regulates protein, lipid, and nucleotide synthesis, whereas mTORC2 is rapamycin-insensitive and regulates cytoskeletal arrangement and pro-survival pathways. Activated T cells derived from the elderly showed higher PI3K/mTOR activity than T cells obtained from younger donors and preferable differentiation towards short-lived T effector cells rather than memory precursors [300]. mTOR has been suggested to serve as a major inducer of a SASP phenotype by elevating expression of MAPK-activated protein kinase 2, which in turn inhibits the RBP ZFP36L1 [274].

Sestrins are induced by p53 [301] and FOXO3 [302], serve as antioxidants, and have been reported to stimulate AMPK via yet unknown mechanisms [303]. Further, sestrins inhibited mTORC1 signaling by binding Rag GTPases that recruit mTORC1 to the lysosome [304]. In line with their mTOR inhibitory effects, sestrins have previously been ascribed anti-aging activity [305]. However, sestrin 2 has also been shown to promote mTORC2 activity by promoting GAP activity towards Rags 2 and AKT Ser/Thr kinase activation by direct interaction [306]. In addition, sestrins simultaneously activate MAPK pathways [307]. MAPKs are signal-transducing kinases that respond to various stimuli, such as mitogens and heat shock and pro-inflammatory cytokines and mediate important cell functions including gene expression, proliferation, differentiation, cell survival, and apoptosis [308,309]. MAPKs comprise three main subgroups, ERK, c-Jun amino-terminal kinases (JNK), and p38 [310]. Lanna et al. observed that sestrins were expressed at a much higher level by senescent CD4^+^CD28^−^ T cells derived from the elderly [311]. Downregulation of sestrin expression by RNA interference largely reversed senescence-associated deficits in senescent T cell populations, including telomerase activity, re-expression of TCR components and CD28, IL-2 production, and proliferative capacity in response to polyclonal activation. These effects were independent of mTOR signaling. In the same study, specifically in senescent T cells sestrins were shown to form a complex with all MAPKs, termed the sestrin–MAPK activation complex (sMAC). Sestrins were required for phosphorylation of AMPK which in turn was necessary for the phosphorylation of MAPK within the sMAC. Of note, pharmacological inhibition of either MAPK in senescent T cells commonly elevated their proliferation. However, each individual MAPK controlled distinct T cell properties. For example, inhibition of JNK restored expression of TCR components and CD28.

## 5. Therapeutics That Counteract Immunosenescence and Inflammaging

In the following, we discuss which compounds have been evaluated to exert anti-aging effects in immune cells on a molecular and cellular level in humans and in preclinical models. Further, we also discuss agents that have been developed primarily for cancer therapy but address targets that play a major role in immunosenescence as well and therefore may be tested in relevant studies. In either case nanoformulations containing the relevant agents may serve to overcome restrictions like poor solubility and passive or even active targeting of distinct cell types may reduce unwanted side effects like toxicity [312]. Furthermore, NCs allow codelivery of compounds with different targets, thereby enabling synergistic effects [313].

In the brain, inflammaging has been acknowledged as an important factor for the development of age-associated neurodegenerative disorders [56] like AD [314], PD [315], and cerebral small vessel disease [316] that may cause intracerebral hemorrhage [317], ischemic stroke [318], and dementia [319]. Most approaches that aim to overcome the blood–brain barrier for drug delivery have focused on receptor-mediated transcytosis, comprising internalization of a therapeutic from the capillary lumen into the brain vasculature and its release into the brain parenchyma [320], and this has been evaluated in a number of preclinical and clinical studies [321]. Conjugation of a receptor-targeting antibody or peptide to drugs has been shown to result in drug accumulation in the brain when addressing the transferrin and insulin receptor [322], respectively, and low-density lipoprotein (LDL) [323] as well as LDL-related protein 1 [324] receptors. So far, most studies have aimed to deliver cytotoxic drugs for treatment of brain tumors [325], but approaches that tackle age-associated neurodegenerative diseases have gained increasing interest as well [326,327]. To date, several nanoformulations have been shown to enable the delivery of drugs to the brain for treatment of age-related disorders, for example, various receptor-targeting liposomes [328] and poly(lactic-co-glycolic acid) (PLGA)-based [329] NCs.

### 5.1. Dietary Supplements

Dietary supplements, including several vitamins, trace elements, and others like omega-3 fatty acids, play important roles in the function of both the innate and adaptive immune system [330]. Older people are often at a higher risk of nutrient deficiency and this can further drive immunosenescence [331].

#### 5.1.1. Micronutrients

The effects of the micronutrients vitamins C, D, and E, zinc, and selenium on immune function and immunosenescence have recently been reviewed [332]. For example, daily oral administration of 500 mg of vitamin C for three months to older people significantly improved functions of PMNs and lymphocytes, including chemotaxis, phagocytosis, and proliferation, close to levels observed for younger adults [333]. Vitamin D exerted several beneficial effects on immunosenescence, for example, promoting a switch of human memory Th17 cells derived from arthritis patients towards IL-10-producing CTLA4^+^ Tregs [334]. Likewise, Rizkaand and coworkers reported on an overall induction of IL-10 in vitro for vitamin D-treated PBMCs derived from older donors [335]. Similar to vitamin C, supplementation of healthy older patients with the antioxidant vitamin E enhanced several immune functions of PMNs, NK cells, and lymphocytes [336]. In old mice, vitamin E supplementation improved NK cell [337] and T cell [338] responses upon influenza infection and PMN-mediated resistance to *Streptococcus pneumoniae* [339]. Supplementation of old mice with zinc expanded the pool of naïve CD4^+^ T cells and dampened the otherwise age-dependently high level of pro-inflammatory cytokines induced by polyclonal in vitro T cell stimulation [340]. Broad beneficial effects counteracting immunosenescence have also been discussed for selenium, which have been attributed largely to its antioxidant activity [341].

Vitamin C [342] and vitamin D [343] also activated SIRT1. SIRT1 has been shown to exert various anti-aging effects on various levels, including the stimulation of DNA repair and inhibition of telomere attrition [344], as well as inflammaging-associated NF-κB activity, promoting autophagy [345], mitochondrial metabolism [346], and FOXO3 expression [347]. SIRT1 requires NAD^+^ as a substrate, which is downregulated in aging due to enhanced expression of the ecto-enzyme CD38 by senescent cells [348]. Accordingly, administration of the NAD^+^ precursors nicotinamide riboside [349] and nicotinamide mononucleotide [350] as well as other dietary compounds has also been shown to promote SIRT1 expression and activity as reviewed in [351].

To date, a large variety of NC types have been evaluated for delivery of nutrients but with a focus on therapeutic traits not associated with immunosenescence. In this regard, nanoformulated vitamin D [352] has been evaluated for treatment of osteoporosis [353] and autoimmune diseases [354] like psoriasis [355], whereas nanoformulations delivering vitamin C [356] and vitamin E [357] in combination with antibodies blocking inhibitory receptors or chemotherapeutics have been tested in tumor therapeutic strategies. A few studies have also shown improved delivery of other dietary supplements like omega-3 fatty acids [358]. Only some studies have included nanoformulations of NAD^+^ precursors to overcome their fast degradation and consequently minor bioavailability of SIRT1. Here, Nie and coworkers showed that orally applied microspheres containing nicotinamide riboside improved its overall bioavailability [359]. Hunt et al. reported on improved cellular uptake of quantum dot-conjugated nicotinamide mononucleotide in the liver after oral administration in old mice, which increased the activity of AMPK and SIRT1 [360].

#### 5.1.2. Autophagy Stimulators

To date, various dietary compounds have been shown to induce autophagy, especially by inhibiting acetyltransferases (e.g., the widespread polyamine metabolite spermidine [361], the polyphenol curcumin derived from *Curcuma longa* [362], and the green tea component epigallocatechin-3-gallate [363]). Alternatively, autophagy may be enhanced by inducing deacetylase activity as shown for the polyphenol resveratrol [364]. All of these agents commonly modulate AMPK/PI3K/mTOR signaling (see Figure 3). Therefore, such nutraceuticals have been suggested to exert anti-aging properties.

##### Resveratrol

Resveratrol has been reported to activate AMPK and thereby limit mTOR [365] and PI3K [366] signaling. Further, resveratrol was demonstrated to induce the autophagy regulators SIRT1 [367] and FOXO1 [368], most probably via AMPK activation [369]. Due to these properties as well as additional diverse anti-tumorigenic modes of action like modulation of glucose metabolism [370] and histone deacetylase activities [364] resveratrol has been tested as an anti-tumor [371] agent, as a therapeutic for other diseases [372], and with regard to anti-aging properties [373]. Of note, resveratrol enhanced the lifespan of aged mice on a high-calorie diet, which was associated with improved insulin sensitivity and elevated AMPK activity [374]. Several studies have also shown a beneficial effect of resveratrol on neurodegenerative diseases, such as AD [375].

Owed to its poor solubility and bioavailability a number of distinct nanoformulations of resveratrol have been assayed for anti-tumor efficacy both in vitro and in vivo as reviewed in [376]. For this, different types of NC systems were used, for example, encapsulating chitosan NCs [377], liposomes [378], and mesoporous silica NCs [379] but also gold NCs that were coated with resveratrol [380].

##### Spermidine

In contrast to resveratrol the polyamine spermidine was demonstrated to enhance autophagy in an SIRT1-independent manner [381] by inhibiting the acyltransferase E1A-binding protein E 300 [361] and histone acetyltransferases [382], enhancing the expression and stability of autophagy-controlling components like TFEB [198] and microtubule-associated protein 1S [383] and promoting AMPK signaling [384], thereby reducing mTOR signaling [361]. Besides its prominent effects on autophagy and AMPK/mTOR signaling spermidine also increased the activity of age-dependently downregulated DNA methyltransferase 3 [385] which counteracted age-dependent upregulation of the **β**2 integrin LFA-1 in human T cells [386] and monocytes [74]. Cellular levels of spermidine decline in an age-dependent manner [387]. Administration of spermidine increased the lifespan of mice [383,388], which was associated with improved autophagy and attenuated oxidative stress [382]. In humans, increased uptake of spermidine via food was associated with lower mortality (NCT03378843) [389]. Spermidine was also demonstrated to reduce neuroinflammation in a murine AD model [390] and to improve cognitive abilities in older adults suffering from dementia [391].

Sermidine-based NPs have been generated to improve polyamine metabolism in tumor cells [392] and to exploit their strong anti-bacterial activity upon topical application [393]. Further, due to their cationic character spermidine and derived spermin have been employed to condense nucleic acids for improved gene delivery into tumor cells [394,395]. More recently, Huang and coworkers demonstrated that spermidine/DNA-based NCs that contained mTOR small interfering (si)RNA alleviated symptoms in a murine acute lung injury model due to synergistic autophagy-promoting effects of the carrier spermidine and its cargo, resulting in M2 polarization of lung macrophages [396].

### 5.2. Signaling Modulators

#### 5.2.1. mTOR Inhibitors

##### Rapamycin

Various mTOR inhibitors have been evaluated for the treatment of age-related diseases [397,398]. They are broadly divided into mTORC1- and mTORC2-specific as well as pan-mTORC inhibitors. Rapamycin, a naturally occurring macrolide produced by *Streptomyces hygroscopius*, inhibits mTORC1 and has been frequently used to evaluate its anti-cancer properties [399]. To overcome limitations in bioavailability and the tolerance-promoting activity of rapamycin upon long-term application [400] the suitability of nanoencapsulated rapamycin has been tested in several preclinical studies. For example, solid lipid NCs loaded with rapamycin and coated with polysorbate for stabilization [401] and PLGA-based NCs containing rapamycin to improve its oral bioavailability [402] performed better than soluble rapamycin in in vitro studies. Nanomicelles composed of a grafted copolymer of polyvinyl caprolactam–polyvinylalcohol–polyethylene glycol (PEG) loaded with the rapamycin derivative everolimus, employed due to its immunosuppressive activity in posterior uveitis, showed better permeation into the cornea than in its soluble form [403]. In preclinical in vivo studies PLGA-based NCs encapsulating rapamcycin and a model antigen conferred antigen-specific tolerance by passively addressing endocytic immune cells [404,405]. Based on the finding that senescent cells overexpress surface CD9 [406], PEGylated liposomes [407] and lactose-coated calcium carbonate NCs [408] were conjugated with CD9-specific antibody, thereby achieving preferential uptake by senescent fibroblasts and, upon loading with rapamycin, they counteracted cell cycle arrest.

##### Rapalogs

Pan-mTORC inhibitors, also known as second-generation mTOR inhibitors, compete with ATP for the active site of the mTOR kinase and are extensively studied as anti-cancer drugs with several drugs currently in clinical trials [409]. However, several rapalogs, i.e., rapamycin derivatives which have a more favorable pharmacokinetic profile, have also been studied with regard to their anti-aging effects [398]. In this regard, Mannick and coworkers reported that in a clinical trial combined application of the dual PI3K/mTOR inhibitor BEZ235 and the mTOR inhibitor everolimus to elderly patients attenuated the rate of observed infections and increased CD4^+^ and CD8+ T cell responses after influenza vaccination, which was accompanied by a decrease in expression of the T cell inhibitory receptor programmed cell death protein (PD)-1 [410].

#### 5.2.2. PI3K Inhibitors

So far, PI3K inhibitors have been scarcely discussed as agents to counteract immunosenescence. However, since mutation-dependent constitutive activation of PI3K signaling contributes to cancer induction and progression [411] several PI3K inhibitors have been approved for tumor therapy, including the pan-PI3K inhibitor copanlisib [412], the PI3K isoform-specific inhibitors alpelisib (PI3Kα) [413], idelasib (PI3Kδ) [414], and duvelisib (PI3Kδ and PI3Kγ) [415], and the dual PI3Kδ and casein kinase 1ε inhibitor umbralisib [416]. In addition, a larger number of further PI3K inhibitors developed to improve efficacy and to lower toxicity are currently being tested in clinical trials as reviewed in [417]. Future studies may evaluate the potential use of PI3K inhibitors for treatment of immunosenescence.

#### 5.2.3. Dual PI3K/mTOR Inhibitors

Besides inhibitors targeting either PI3K or mTOR for tumor therapy, a number of dual PI3K/mTOR inhibitors developed to counteract treatment-induced resistance are currently being tested in phase 1 and 2 clinical trials, comprising apitolisib [418], bimiralisib [419], gedatolisib [420], paxalisib [421], and samotolisib [422]. In preclinical studies, the dual PI3K/mTOR inhibitor BEZ235 was encapsulated into CaCO_3_ nanocrystals for T cell lymphoma treatment [423] and into the hydrophobic PLGA core of diblock copolymers conjugated with B cell-targeting antibodies for B cell lymphoma treatment [424] to overcome its poor solubility and toxicity.

##### Metformin

The first-line anti-diabetic drug metformin [425] has also been demonstrated to inhibit PI3K and mTOR [21,426] signaling at least in part via AMPK activation [427]. Its numerous distinct positive effects on metabolic pathways have been reviewed in [428]. Effects of metformin on the immune compartment have been elucidated primarily in the context of tumor therapy. Here, metformin was shown to exert anti-tumor effects on several levels, including the reprogramming of cells within the tumor microenvironment via activation of AMPK and by inhibition of STAT3 [429] and NF-κB [430]. In agreement, metformin inhibited the SASP phenotype of senescent cells by preventing nuclear translocation of NF-κB and activating NF-κB inhibitor α in an AMPK-independent manner [431]. In addition, metformin inhibited MDSC activity by inhibiting expression of programmed cell death protein ligand (PD-L)1 [432] and of the ATP-degrading ectoenzymes CD39 and CD73 [433] in an AMPK-dependent manner and ROS production via FOXO3 induction [434]. Further, metformin stimulated CTL activation, including stronger IFN-γ and TNF-α production in response to stimulation, via metabolic reprogramming, by stimulating glycolysis and under low-glucose conditions via fatty acid oxidation and autophagy-dependent glutaminolysis [432]. Moreover, metformin inhibited Foxp3-dependent Treg induction by activating AMPK, which in turn inhibited the Foxp3 regulator mTORC1 [435]. So far, the anti-tumor effects of metformin have been assessed in a number of clinical trials, albeit with controversial outcomes [436]. All of the aforementioned effects are also able to counteract immunosenescence. However, the efficacy of metformin as an anti-aging drug is still under debate and further clinical trials are needed to substantiate the preliminary beneficial findings obtained in the course of clinical studies [437]. Several studies have demonstrated a correlation between type 2 diabetes mellitus and AD, indicating a potential overlap in the underlying pathophysiological mechanisms. The potential use of metformin for AD treatment has been reviewed by Khezri et al. [438].

Nanoformulated metformin may overcome limited bioavailability and enable cell targeting. In agreement, in a number of in vivo diabetes models [439,440] but also in vitro tumor cell studies [441,442] and some in vivo mouse tumor studies **[443,444]** encapsulated metformin exerted stronger effects than in its soluble form. So far, a variety of NCs have been employed for nanoformulation of metformin, quite often polymeric carriers [442], nanoemulsions [440], and liposomes [444]. Again, the suitability of nanoformulated metformin for treatment of immunosenescence has been scarcely addressed so far. Hunt and coworkers reported that in mice quantum dot-conjugated metformin after oral uptake accumulated in the liver and yielded stronger AMPK and SIRT1 activation in hepatocytes and liver sinusoidal endothelial cells by several orders of magnitude than soluble metformin [360]. Further, hepatocytes derived from old mice internalized nanoformulated metformin to a higher extent than when applied in its soluble form. Whereas cellular uptake of soluble metformin is mediated by organic cation transporters [445], nanoformulated metformin was endocytosed in a clathrin-dependent manner [360]. The authors concluded that the alteration of the uptake route compensated in part for the overall reduced endocytic activity of hepatocytes of old mice.

##### NP-Intrinsic mTOR-Regulating Function

Interestingly, NCs themselves may modulate mTOR activity. Activated mTORC1 localizes to the outside of the membrane of lysosomes, controlling autophagy in the lysosome, and in turn is regulated by various factors, including arginine, within the lysosome [446]. Of note, polystyrene NCs with amino groups that accumulated in lysosomes have been reported to increase the lysosomal pH value and thereby to inhibit mTOR signaling, whereas carboxylated NCs stimulated mTOR activity [447]. These findings underscore that intrinsic properties of NCs need to be tested in the course of designing functionalized derivatives and that such properties may even be exploited.

### 5.3. Selective Inhibition and Killing of Senescent Cells

Senescent cells that are characterized by replicative arrest express p16^Ink4a^ and accumulate in the elderly due to an impaired turnover [11] caused in part by upregulation of the non-classical MHC receptor human leukocyte antigen (HLA)-E in a SASP-dependent manner [448]. HLA-E engages the inhibitory receptor NKG2 expressed by NK cells [449] and CTL [450], thereby avoiding immune clearance. Besides aging-associated factors like genomic instability [451] a variety of exogenous stress factors like treatment with cytotoxic drugs as used in tumor therapy [452] but also viral infections like SARS-CoV-2 [453] promote the induction of senescent cells. Senescent cells are characterized by cell cycle blockade and apoptosis arrest via upregulation of survival anti-apoptotic pathways involving BCL-2 members, p53, and the cyclin-dependent kinase inhibitor of p16^Ink4a^ and p21. On the one hand, cellular senescence has been considered as a mechanism to limit cell proliferation and to prevent malignant transformation of cells with genomic instability [454] that is spread to neighboring cells by SASP [455] and to support wound healing by platelet-derived growth factor AA [456]. On the other hand, a number of studies have shown that senescent cells are involved in (age-dependent) diseases including cancer in part via continuous low-level inflammation [457]. Moreover, Baker and coworkers demonstrated that inducible deletion of p16Ink4a-expressing senescent cells in aged mice counteracted age-dependent decreases in organ function and increased their lifespan [458]. Therefore, as outlined in the following based on library screenings a number of pharmacological agents that kill senescent cells, termed senolytics, and immune-related approaches aimed to delete senescent cells have been developed.

#### 5.3.1. Senolytics

##### Dasatinib/Quercetin

The dual Abelson interactor (ABL)/sarcoma (SRC) tyrosine kinase inhibitor dasatinib is clinically used for treatment of chronic myeloid leukemia that is characterized by constitutively active breakpoint cluster region–ABL activity [459]. The polyphenol quercetin was reported to exert anti-inflammatory and anti-oxidant activities [460] and has also been reported to show tumor-inhibiting effects in part due to modulation of the PI3K and MAPK signaling axis [461]. A combination of both agents was shown to effectively kill senescent cells [462] and thereby revealed the role of senescent cells for the progression of various diseases in different organs.

For example, in a mouse model of idiopathic pulmonary fibrosis the frequency of senescent fibroblasts was enhanced, and their depletion by combined administration of dasatinib and quercetin improved pulmonary health [463]. Aging constitutes a risk factor for non-alcoholic fatty liver disease [464]. Ogodnik and colleagues showed that disease progression was correlated with an increase in senescent hepatocytes, which was counteracted by application of dasatinib/quercetin [465]. In aged mice, depletion of senescent cells by treatment with dasatinib/quercetin reduced levels of pro-inflammatory mediators in the intestine and altered the composition of the gut microbiome [466]. Further, infection of old mice with a mouse β-coronavirus that resulted in an expansion of senescent cells and was lethal in most cases could be counteracted by treatment with dasatinib/quercetin [467]. Furthermore, Xu and colleagues demonstrated that injection of preadipocytes that were irradiated beforehand to induce senescence exerted deleterious effects on physical fitness [468]. Combined treatment with dasatinib and quercetin improved these parameters and prolonged the lifespan of young mice preinjected with senescent cells as well as of aged mice.

##### BCL-2 Family Inhibitors

To date, several agents have been described to exert senolytic activity and many of these act by inhibiting members of the BCL-2 family that exert anti-apoptotic functions. In this regard, navitoclax, which inhibits BCL-2 and BCL-xL [469], was reported to deplete various types of senescent cells, including senescent HSCs, in aged mice as well as in mice irradiated to promote cellular senescence, thereby rejuvenating the HSC compartment [470]. Senolytic activity has also been demonstrated for the flavone fisetion, known to target the same BCL-2 family members as navitoclax [471] and two BCL-xL-specific inhibitors (A1331852, A1155463) that were considered favorable due to low toxicity [472].

##### Other Agents

The curcumin analog EF24 has been shown to inhibit BCL-2 and other anti-apoptotic proteins like X-linked inhibitor of apoptosis and baculoviral IAP repeat-containing (Birc)2 and Birc7, but also NF-κB [473], and to modulate miRNA expression and proteasomal activity [474]. EF24 exerted senolytic activity, which was synergistically enhanced upon coadministration of navitoclax [475]. Senolytic activity has also been shown for the histone deacetylase (HDAC) inhibitor panobinostat [476] and the dual HDAC/PI3K inhibitor CUDC907 [477] shown to decrease BXL-xL (panobinostat) and Bcl-2 homology domain 3 (CUDC-907), respectively, and that have been applied for treatment of multiple myeloma [478,479]. The members of the bromodomain and exterminal domain family (BET) regulate expression of inflammation-associated genes with acetylated H3 and H3 histones by recruiting various transcription factors [480]. Hence, BET activity has been linked to SASP induction [481]. Wakita et al. reported that BET protein degrader exerted senolytic activity but identified attenuated DNA repair and enhanced expression of autophagy-related genes as causative [482]. Additionally, inhibitors of heat shock protein 90, in part via upregulation of autophagy [483] and the polyphenol procyanidin C1 in a ROS-dependent manner [484], deleted senescent cells and prolonged survival in a mouse model of progeroid syndrome and of aged mice, respectively. The efficacy of senolytics to improve treatment of various diseases has prompted a number of clinical trials (phases 1–2) employing most often dasatinib/quercetin and fisetin, respectively, as recently reviewed in [485]. Most of these studies are still ongoing.

A number of studies have reported on NCs functionalized with senolytics that killed senescent cells in vitro, but due to their non-selective targeting properties their therapeutic usage may be limited [486]. Pronounced lysosomal β-galactosidase activity is a hallmark of senescent cells [487]. Encapsulation of drugs into galactose-coated NCs [488] and the administration of galactose-modified prodrugs [489,490] has been shown to enable targeting of senescent cells and to improve specific killing of such cells, thereby minimizing adverse effects. Xu and coworkers demonstrated that NCs targeting β-2-microglobulin (B2M), found overexpressed by senescent cells [491], preferentially engaged senescent cells in vitro and in vivo in aged mice [492]. NCs encapsulating dasatinib specifically killed senescent cells, whereas no cytotoxic activity was observed when applying dasatinib to the cell culture at an equimolar concentration.

#### 5.3.2. Biologicals and Immunotherapeutic Approaches

As an alternative to pharmacological inhibitors various biologicals have also been developed to eliminate senescent cells. Based on the finding that the TF FOXO4 and p53 interact to promote p21 expression [493], FOXO4-based peptides were developed that prevented FOXO4/p53 interaction, thereby reducing senescent cells in mice and enhancing physiological fitness and organ function in aged mice [494]. Poblocka and coworkers showed that a B2M-specific antibody conjugated with the cytotoxic drug duocarmycin killed senescent cells in vitro [495], but further experiments are necessary to evaluate the specificity of this approach in vivo. As an alternative approach, immunization against proteins strongly upregulated by senescent cell populations to induce immune responses directed against these cells has been successfully tested in mice. Yoshida et al. showed in a model of obesity-dependent accumulation of CD153^+^ senescent T cells in visceral adipose tissue that CD153-specific immunization resulted in the depletion of CD153-expressing senescent T cells and conferred glucose tolerance [496]. Immunization of mice against glycoprotein non-metastatic melanoma protein B (GPNMB) that is highly expressed by senescent endothelial cells and leukocytes resulted in specific depletion of GPNMB^+^ cells associated with a decrease in aging-associated organ phenotype and prolonged the lifespan of progeroid mice [497].

## 6. Inhibition of Immunoregulatory Cell Types

**Figure 4:**
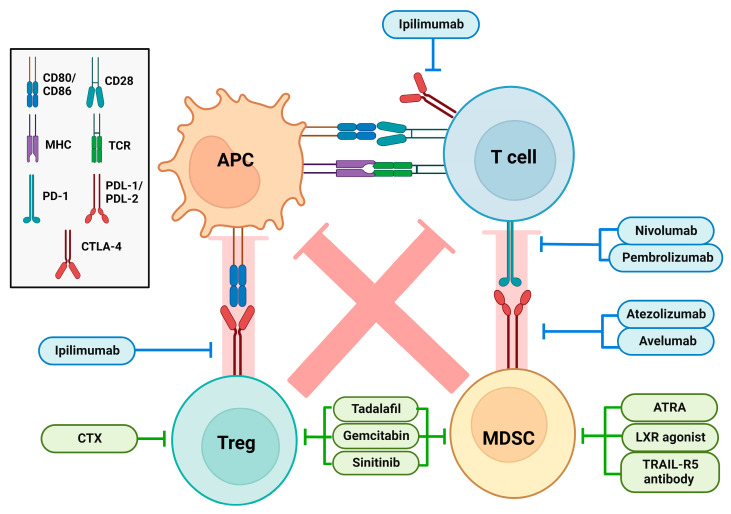
Aging is associated with an increase in immunoregulatory Tregs and MDSCs. Their number and activity can be blocked by immune checkpoint inhibitors (e.g., ipilimumab, nivolumab, pembrolizumab, atezolimumab, avelumab) and various pharmacological agents used for anti-tumor therapy. LXR = liver X nuclear receptor; see list of abbreviations.

Several aspects of age-dependent immune dysregulation are reminiscent of the immunocompromised state of tumor patients [498]. Here, both the tumor and tumor-associated (immune) cells that collectively form the tumor microenvironment (TME) [499] generate factors that serve to differentiate and activate Tregs and MDSCs [500]. These immunoregulatory cell populations inhibit the activity of innate and adaptive immune cells throughout the body, whereas the immunosuppressive microenvironment of the TME inhibits the functional activity of tumor-infiltrating immune effector cells. In the elderly, the frequency of immunoregulatory MDSCs [501] and Tregs [502] was found increased as well. Counteracting the immunosuppressive effects of tumor/TME-expanded and -induced MDSCs and Tregs is therefore a major goal of immunotherapeutic tumor strategies and a number of relevant agents have been clinically approved as depicted in Figure 4.

### 6.1. Immune Checkpoint Inhibitors

Immunoregulatory cells comprising MDSCs, Tregs, and tolerogenic APCs but also tumor cells display surface receptors which engage counter-receptors of APCs and T cells and by subsequently induced signaling inhibit their functions [503], termed immune checkpoints. Immune checkpoint inhibitors (ICIs) constitute surface receptor-binding antibodies that block the activity of such inhibitory receptors.

APCs costimulate antigen-specific T cells via CD80 and CD86 that bind CD28 on the T cell [504]. CTLA-4 is highly expressed by Tregs and engages costimulatory receptors on APCs with a stronger affinity than CD28 [505]. This prevents on the one hand binding and costimulation of contacting naïve antigen-specific T cells and on the other hand induces inhibitory signaling within the APC [506]. The CTLA-4-blocking antibody ipilimumabwas first approved for melanoma treatment and subsequently for other types of tumors [507]. PD-1 is apparent on T cells and engagement of its ligands programmed cell death 1 ligand (PD-L)1 and PD-L2, which are broadly expressed by tumor cells, MDSCs, and tolerogenic DCs, yields inhibitory signaling in T cells [508]. So far, anti-PD-1 (nivolumab, pembrolizumab) and PD-L1 (atezolizumab, avelumab)-binding antibodies have been approved for cancer therapy [503].

In two consecutive studies Henson and coworkers demonstrated that blockade of CTLA-4, PD-L1, and PD-L2 on human CD8^+^ T cells derived from the elderly [509] as well as inhibition of PD-1 on human senescent effector memory CD8^+^ T cells [510] enhanced CD8^+^ T cell proliferation in response to polyclonal stimulation. Nonetheless, so far effects of clinically approved ICIs on immune functions in the elderly have been studied primarily in the context of tumor therapy [511]. As concluded from a meta-analysis ICIs also worked well in aged patients (>75 years) when applied as a first-line treatment [512,513].

In a number of chronic viral infections that may also promote age-associated immunosenescence like EBV [514], hepatitis B virus (HBV) [515], and human immunodeficiency virus (HIV-)1 [516] elevated expression of immune checkpoints was observed on virus-specific T cells and has been associated with an exhausted/senescent phenotype [517,518,519]. Interestingly, CMV-specific CD8^+^ T cells displayed no enhanced expression of coinhibitory receptors found upregulated in immunoregulatory cell types and exhausted T cells, respectively [520]. However, CMV-specific terminally differentiated effector CD8^+^ T cells were characterized by enhanced expression of the inhibitory receptor CD85j [521]. CD85j engages widely expressed MHCI alleles and inhibits cell activation by SHP-1 recruitment [522,523]. Antibody-mediated blockade of CD85j on CMV-specific CD8^+^ T cells enhanced their proliferative activity [521].

In the case of HIV-1 several case reports demonstrated an increase in HIV-1 levels in response to treatment with immune checkpoint inhibitors that targeted CTLA-4 or the PD-1/PD-L1 axis [524]. In preclinical studies, inhibition of this signaling axis increased HBV-specific T cell responses, whereas in the few clinical trials performed so far no major success was observed [525]. In the case of EBV, in a fully humanized mouse model administration of the PD-1-blocking antibody pembrolizumab elevated EBV spread [526].

Since only a fraction of tumor patients display long-lasting responses towards ICIs, partially due to compensatory upregulation of other immune checkpoints [527], the efficacy of a number of blocking antibodies for several alternative immune checkpoints is currently being tested in clinical trials focusing on the suitability of ICI combinations [503]. For example, concurrent blockade of lymphocyte-activation gene 3 using relatlimab in combination with nivolumab yielded superior results as compared with nivolumab monotherapy (NCT03470922) [528]. Similar findings were obtained in a number of clinical trials assessing concomitant blockade of T cell immunoglobulin and ITIM domain and the PD-1/PD-L1 signaling axis [529].

### 6.2. Inhibition of Immunoregulatory Cells

Besides ICIs drugs that deplete or reprogram MDSCs and Tregs to overcome their inhibitory activity are also currently being evaluated. Here, clinical trials demonstrated that orally applied metronomic cyclophosphamide (CTX) resulted in selective depletion of Tregs in cancer patients [530]. The mTOR inhibitor everolimus is applied due to its direct tumoricidal effects [531] but was reported to promote Treg expansion due to Foxp3 induction as observed, for example, in accordingly treated patients after liver transplantation [532]. However, cotreatment of metastatic renal cell carcinoma patients with everolimus and CTX in a phase 1 trial (NCT01462214) yielded Treg depletion and unexpectedly also a decrease in MDSC numbers [533].

Phosphodiesterase (PDE)5 inhibitors were shown to inhibit the immunosuppressive functions of MDSCs by downregulating expression of nitric oxide synthase (NOS)2 and arginase-1 [534]. Application of the PDE5 inhibitor tadalafil [535] lowered the frequency of MDSCs, but unexpectedly also that of Tregs, in head and neck squamous cell carcinoma patients (NCT00843635) [536].

Further, cancer patients treated with the pyrimidine nucleoside prodrug gemcitabine, which after incorporation into genomic DNA interferes with cell replication, were reported to display a reduced number of granulocytic (g)MDSCs and Tregs [537]. Likewise, the tyrosine kinase inhibitor sunitinib that is frequently used for treatment of metastatic renal cell carcinoma (mRCC) [538] also reduced MDSC [539] and Treg [540] numbers in patients at least in part by interfering with c-kit signaling [541].

All-trans-retinoic acid (ATRA) has been reported to overcome the differentiation block of MDSCs [542]. In this line, in a number of clinical trials have administered ATRA either alone (mRCC; NCT00100906) [543] or in combination with other agents, for example, pembrolizumab (melanoma; NCT03200847) [544] reduced MDSC numbers in cancer patients. However, since ATRA also promoted Treg induction [545], it is conceivable that combination therapies including a Treg-inhibiting agent as assessed for ipilimumab (melanoma; NCT02403778) [546] may be favorable to overcome the inhibitory activity of either immune-inhibitory cell type.

As demonstrated in an extensive study by Tavazoie and coworkers agonists of the liver-X nuclear receptor induced apoptosis specifically in MDSCs both in mouse tumor models and in a subsequently performed clinical study of advanced cancer patients (NCT02922764) [547]. Tumor cells and MDSCs display high expression of the TNF-related apoptosis-induced ligand receptor (TRAIL-R)5 [548] which upon engagement, e.g., by TRAIL on T effector cells, induced apoptosis [549]. In agreement, in a clinical study treatment of cancer patients with agonistic TRAIL-R5 antibody (NCT02076451) resulted in a strong reduction of MDSCs due to direct interaction with these cells [550,551].

Wiktorin and coworkers demonstrated in different tumor mouse models that the NADPH oxidase 2 inhibitor histamine dihydrochloride (HDC) attenuated MDSC numbers [552]. Based on these data patients with acute myeloid leukemia (AML) were treated with HDC and IL-2 (NCT01347996), which resulted in a decrease in monocytic (m)MDSCs.

The anti-CD33 toxin conjugate gemtuzumab ozogamin is clinically applied for treatment of CD33-positive AML [553]. Fultang and coworkers (2019) reported that gMDSCs and (m)MDSCs derived from cancer patients expressed CD33 as well and that in vitro treatment of patient-derived MDSCs with gemtuzumab ozogamin efficiently killed MDSCs [554].

Elevated STAT3 activity is a hallmark of many tumors [555] but also myeloid immunoregulatory cell types including MDSCs [556] as well as Tregs [557]. Therefore, a number of STAT3-targeting inhibitors are currently being tested in a number of clinical trials [558], often in combination with ICIs, for example, the STAT3-specific small interfering (si)RNA danvatirsen and the PD-L1-specific antibody durvulamub (NCT02983578).

Future studies are necessary to clarify whether ICIs and drugs that act on MDSCs and Tregs may improve immune responses in the elderly and the success of vaccination. Here, the possibility of immune-related adverse effects that may arise upon treatment with such agents needs to be taken into account. However, cell-targeted delivery by nanoformulations as evaluated in numerous preclinical studies may strongly decrease such side effects [559] as evidenced in the case of clinically approved nanoformulated chemotherapeutics [560].

## 7. Vaccine Formulations

Impaired responses of the elderly towards vaccinations as shown, for example, for influenza vaccines based on attenuated/killed virus [561] but also towards recently developed mRNA-based severe acute respiratory syndrome coronavirus type 2 (SARS-CoV-2) vaccines [562,563] have been attributed to impaired stimulatory responsiveness and T cell stimulatory activity of APCs [94], lowered numbers of naïve T cells [300], and B cells [564,565] characterized by impaired responsiveness [226]. B cell responses may also be hampered by attenuated Th [566] and Tfh [567] activity. Moreover, as outlined above, chronic viral infections, for example, with CMV that affects around 60% [568] to more than 80% [569] of people worldwide, have been repeatedly reported to contribute to immunosenescence. In the case of CMV the sustained expansion of CMV antigen-specific CD8^+^ T cells, which can constitute up to about 25% of the total CD8^+^ T cell compartment in the elderly [570], termed memory inflation [571], can indirectly inhibit the activation of other T cells [572,573]. In contrast to T cells, however, latent CMV infection may not compromise vaccination-induced humoral immune responses [520].

Besides defects in APC activity and the stimulation of adaptive immune cells, increased numbers in immunoregulatory MDSCs [501] and Tregs [127,131,132] may affect the extent and shape of vaccination-induced immune responses. Finally, age-associated inflammaging, which results in constitutive triggering of danger receptors, also attenuates the responsiveness of immune cells towards vaccination-associated adjuvants [574].

As exemplified by influenza vaccines, the attenuated response in the elderly was shown to be overcome by enhancing the dose of antigen [575], intradermal instead of intramuscular application [576] to enable direct vaccine uptake by skin DCs [577], and the inclusion of adjuvants [578]. So far, these adjuvants are squalene-based water-in-oil emulsions (e.g., MF59, AS03, and AF03) [579] and aluminum salts [580] that stimulate immune reactions on several levels.

Ongoing immunotherapeutic approaches in tumor research focus on the development of DC-targeting nanovaccines that allow physical codelivery of tumor antigens and a DC-stimulatory adjuvant to mount sustained (primary) T cell responses [581]. Most DC populations load peptides derived from exogenous antigen onto MHCII complexes recognized by CD4^+^ T cells [582]. However, cDC1 redirects part of the internalized protein or peptide antigen to enable efficient loading of MHCI complexes for CD8^+^ T cell stimulation to induce antigen-specific CTL [583]. Therefore, cDC1 with its pronounced cross-presenting potential is of high interest for DC-targeting vaccines [584,585]. In many cases these nanovaccines are also applied intradermally to address skin DCs and to enable transfer of the vaccine to skin-draining lymph nodes [586,587]. Alternatively, vaccines may be applied systemically to achieve fast distribution of the vaccine in secondary lymphoid organs, as discussed, especially in the context of cancer immunotherapy [588]. Similar approaches might also be advantageous for the development of nanovaccines in the context of infectious diseases to overcome impaired APC activity and defects in the B cell compartment in the elderly.

Several types of NC have been demonstrated to bind APCs by so-called passive targeting. Here, APC binding is mediated most often by the protein corona that coats the NC surface and consists of distinct serum factors [589], which in turn often constitute ligands of scavenger receptors [590] and MAC-1 [591] that are strongly expressed by myeloid cell types. Besides passive adsorption of serum factors, the NC surface may also be recognized by soluble components of the innate immune system, comprising antibodies and complement factors (see below). Dense decoration of NCs with PEG largely prevents the formation of a protein corona [592]. Kranz and coworkers demonstrated that formulations of PEGylated liposomes that varied in size and charge also differed in organ accumulation and cellular binding and delineated one derivative with pronounced DC-specific binding activity [593,594]. Such liposomes containing tumor antigen-specific mRNA successfully induced strong and sustained anti-tumor T cell responses in melanoma patients (NCT02410733) [595].

However, in most ongoing studies DC-specific targeting is achieved by the decoration of nanovaccines with an antibody specific for a surface receptor predominantly expressed by DCs, for example, C-type lectin domain-containing (CLEC-)9a [596] or a derivative of a natural ligand like the carbohydrate trimannose reported by us and others to bind the C-type lectin receptor DC-specific intercellular adhesion molecule-3-grabbing non-integrin to a high extent [597,598].

So far, the vast majority of nanovaccines have been designed to target DCs [599]. Somewhat surprisingly, however, B cells, as the inducers of humoral immune responses that are highly important for the neutralization of pathogens and the killing of infected cells, have not been the focus of research. We have shown that NCs coated with carbohydrates to facilitate biocompatibility triggered the lectin-dependent complement pathway which resulted in opsonization of the carrier with active complement and subsequently in uptake by B cells via CR1/2 [600]. To some extent these NCs engaged inflammatory DCs, monocytes/macrophages, and neutrophils via MAC-1 [601], also termed complement receptor (CR)3 [602]. Most interestingly, these NCs, when coated with Th1 response-promoting immunostimulatory CpG oligonucleotides (TLR-9 ligand) plus a model antigen, strongly activated target cells and induced a stronger antigen-specific cellular and humoral immune response in mice as compared to immunizations using equimolar amounts of soluble antigen and adjuvant [600]. In therapeutic settings, this B cell-directed targeting approach prevented airway hyper-responsiveness and anaphylactic shock reactions by inducing a Th1 immune response associated with IgE inhibition.

It is conceivable that systemic application of nanovaccines that directly target B cells may also yield a more efficient antibody response in the elderly as compared to topically applied conventional vaccines. In the aforementioned study, we also observed uptake of the complement-opsonized functionalized NCs by follicular dendritic cells via CR1/2. FDCs store protein antigen for long periods of time and are crucial for B cell maturation, antibody isotype class switching, and the generation of B memory cells [603]. Therefore, further studies should focus on determining the induction of long-term immunity by this approach.

Altogether, further studies are necessary to assess the suitability of DC- and B cell-addressing nanovaccines to overcome the compromised state of APCs and adaptive immune cells in the elderly in order to induce strong pathogen-specific immune responses.

## 8. Conclusions

Chronic viral infections [8], cancer [208], and aging [1] commonly affect the immune system and cause overlapping patterns of immunosenescent features. Especially in cancer therapeutic drugs and derived nanoformulations have been established to counteract tumor immune evasion on several levels that may also prove successful to overcome immunosenescence.

For example, as outlined above, tumor-induced circulating immunoregulatory cell types comprising Tregs and MDSCs that inhibit both innate and adaptive immune responses [83] are also expanded in persisting infections [604,605] and in the elderly [85,127]. Hence, agents like ICIs that impair the immunoregulatory activity of these cells (see Section 6.1) and pharmaceuticals that attenuate their expansion that have been clinically tested in cancer patients (see Section 6.2) may also be employed to improve immune responses in the elderly. The finding that Tregs inhibit telomerase activity in other leukocytes and thereby promote cellular senescence [167,168] suggests that anti-aging therapies may focus on distinct cell types that play a key role in overall regulation of the leucocyte compartment. In general, the age-dependently increasing number of senescent cells may be effectively reduced by application of senolytics [485].

The attenuated responsiveness of the elderly towards conventional vaccination strategies (see Section 7) may be overcome on the one hand by (partial) inhibition of the expanding immunoregulatory cell compartment as well as currently clinically tested vaccination strategies that directly address DCs to evoke T cell-focused anti-tumor responses [594,595] as well as preclinically evaluated approaches for direct B cell targeting to induce stronger antibody responses [600].

As a further perspective, profound knowledge on molecular causes of immunosenescence, for example, dysregulated non-coding RNA and TF expression as discussed in Section 3.3, may enable nano-based strategies that aim to restore molecular function by using specific siRNA as a cargo. Such approaches have been successfully evaluated in clinical trials for cancer therapy [606]. In addition, metabolic reprogramming of exhausted/senescent T cells [607,608] may yield synergistic effects.

Taken together, the development of strategies to improve healthy aging has become an important issue due to the ongoing increase in life expectancy. As outlined in this review several treatment strategies developed for cancer therapy may also prove successful when “repurposed” for anti-aging applications. Combined administration of such drugs with more established anti-aging compounds like metformin [430] and resveratrol [374,375] as well as senolytics [455] may counteract (immuno)senescence on various levels and thereby achieve synergistic effects. By this approach the onset of age-related disorders that are associated with chronic inflammation [609], for example, cancer [457,610] and autoimmune [611,612], cardiovascular [613], liver [614], and neurodegenerative [314] diseases, may be strongly attenuated, thereby extending the time span of healthy aging and reducing the costs of medical care in aging societies.

## Data Availability

Not applicable.

## Abbreviations

ABC	Age-associated B cell
AD	Alzheimer’s disease
AMPK	AMP-activated protein kinase
APC	Antigen-presenting cell
ATAC-seq	Assay identifying transposase-accessible chromatin by sequencing
ATRA	All-trans-retinoic acid
B2M	Β-2-microglobulin
BACH2	BTB domain and CNC homolog 2
BCL6	B cell lymphoma 6
BET	Bromodomain and exterminal domain family
BIRC	Baculoviral IAP repeat-containing
CCR2	C-C chemokine receptor type 2
CCL	C-C motif chemokine ligand
CD	Cluster of differentiation
cDC	Conventional DC
CMV	Cytomegalovirus
c-Myc	Cellular myelocytomatosis
COX-2	Cyclooxygenase 2
CR	Complement receptor
CTL	Cytotoxic T lymphocyte
CXCL	C-X-C motif chemokine ligand
DAMP	Damage-associated molecular pattern
DC	Dendritic cell
EBV	Epstein–Barr virus
Ercc	Excision repair cross-complementation group
ERK	Extracellular signal-regulated kinase
FOXO1	Forkhead box protein O1
GPNMB	Glycoprotein non-metastatic melanoma protein B
GM-CSF	Granulocyte–macrophage colony-stimulating factor
gMDSC	Granulocytic myeloid-derived suppressor cell
HBV	Hepatitis B virus
HDAC	Histone deacetylase
HDC	Histamine dihydrochloride
HIF	Hypoxia-induced factor
HIV-1	Human immunodeficiency virus
HLA-E	Human leukocyte antigen E
HSC	Hematopoetic stem cell
ICI	Immune checkpoint inhibitor
Ig	Immunoglobulin
iNOS	Inducible nitric oxide synthase
IFN	Interferon
IL	Interleukin
JNK	c-Jun amino-terminal kinase
LDL	Low-density lipoprotein
LFA-1	Lymphocyte function-associated antigen 1
IRF	Interferon regulatory factor
KC	Kupffer cell
KDM4	Lysine (K)-specific demethylase 4
LPS	Lipopolysaccharide
MAC-1	Macrophage-1 antigen
MAPK	Mitogen-activated protein kinase
MDSC	Myeloid-derived suppressor cell
MHC	Major histocompatibility complex
miRNA	MicroRNA
mMDSC	Monocytic MDSC
mRCC	Metastatic renal cell carcinoma
NC	Nanocarrier
NET	Neutrophil extracellular trap
NF90	Nuclear factor of activated T cells 90 KDa
NF-κB	Nuclear factor κ-light-chain-enhancer of activated B cells
NK cell	Natural killer cell
NOS	Nitric oxide synthase
PD-L1	Programmed cell death protein ligand 1
PBMC	Peripheral blood mononuclear cells
PD	Parkinson’s disease
PMN	Polymorphonuclear neutrophilic leukocyte
PRDM1	PR domain-containing 1, with ZNF domain
miRNA	MicroRNA
mTOR	Mammalian target of rapamycin
mTORC	mTOR complex
Mzi-1	Myc-interacting zinc finger protein 1
NLRP3	NOD-like receptor family pyrin domain-containing 3
NOD	Nucleotide-binding oligomerization domain
PAX	Paired box
PD-1	Programmed cell death protein 1
PDE	Phosphodiesterase
PD-L1	Programmed cell death 1 ligand 1
pDC	Plasmacytoid DC
PEG	Polyethylene glycol
PI3K	Phosphoinositide 3-kinase
RBP	RNA-binding protein
ROS	Reactive oxygen species
RUNX3	Runt-related transcription factor 3
SARS-CoV-2	Severe acute respiratory syndrome coronavirus type 2
SASP	Senescence-associated secretory phenotype
SHP-1	Src homology domain-containing protein tyrosine phosphatase-1
siRNA	Small interfering RNA
SIRT1	Sirtuin 1
SPI1	Spleen focus forming virus pro-viral integration oncogene 1
SOCS	Suppressor of cytokine signaling
STAT	Signal transducer and activator of transcription
TCF1	T cell factor 1
TERC	Telomerase RNA component
TERT	Telomerase reverse transcriptase
TF	Transcription factor
Tfh	Follicular helper T cell
TGF-β	Transforming growth factor β
TLR	Toll-like receptor
TME	Tumor microenvironment
TNF-α	Tumor necrosis factor-α
TRAIL-R5	TNF-related apoptosis-induced ligand receptor 5
Th	T helper cell
Treg	Regulatory T cell
WIP1	Wiskott–Aldrich syndrome protein-interacting protein 1
ZFP36L1	Zinc finger protein 36, C3H type-like 1
